# Correction to: Effective machine-learning assembly for next-generation amplicon sequencing with very low coverage

**DOI:** 10.1186/s12859-019-3318-z

**Published:** 2020-01-22

**Authors:** Louis Ranjard, Thomas K. F. Wong, Allen G. Rodrigo

**Affiliations:** The Research School of Biology, The Australian National University, Canberra, Australia

**Correction to: BMC Bioinform**

**https://doi.org/10.1186/s12859-019-3287-2**

Following publication of the original article [1], the author reported that there are several errors in the original article;

1. The figures’ order in HTML and PDF did not match with each other.
  In the original article incorrect Fig. 3 was the correct Fig. 7.
  In the original article incorrect Fig. 4 was the correct Fig. 6.
  In the original article incorrect Fig. 5 was the correct Fig. 3.
  In the original article incorrect Fig. 6 was the correct Fig. 4.
  In the original article incorrect Fig. 7 was the correct Fig. 5
2. The caption of Table 1 was published incorrect.

**Fig. 1:**
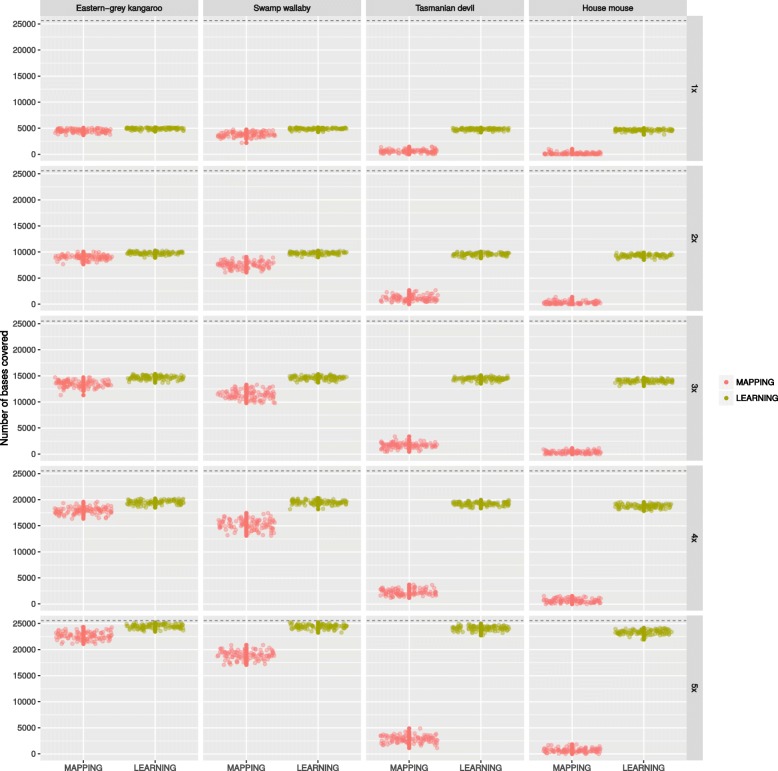
Realised coverage obtained by mapping (MAPPING) or aligning (LEARNING) sequencing reads to increasingly distant homologous reference sequences. The short-reads originate from a western-grey kangaroo amplicon of length 5,130bp with 5× coverage, therefore the expected number of bases covered is ∼25, 000 (dashed line)

**Fig. 2:**
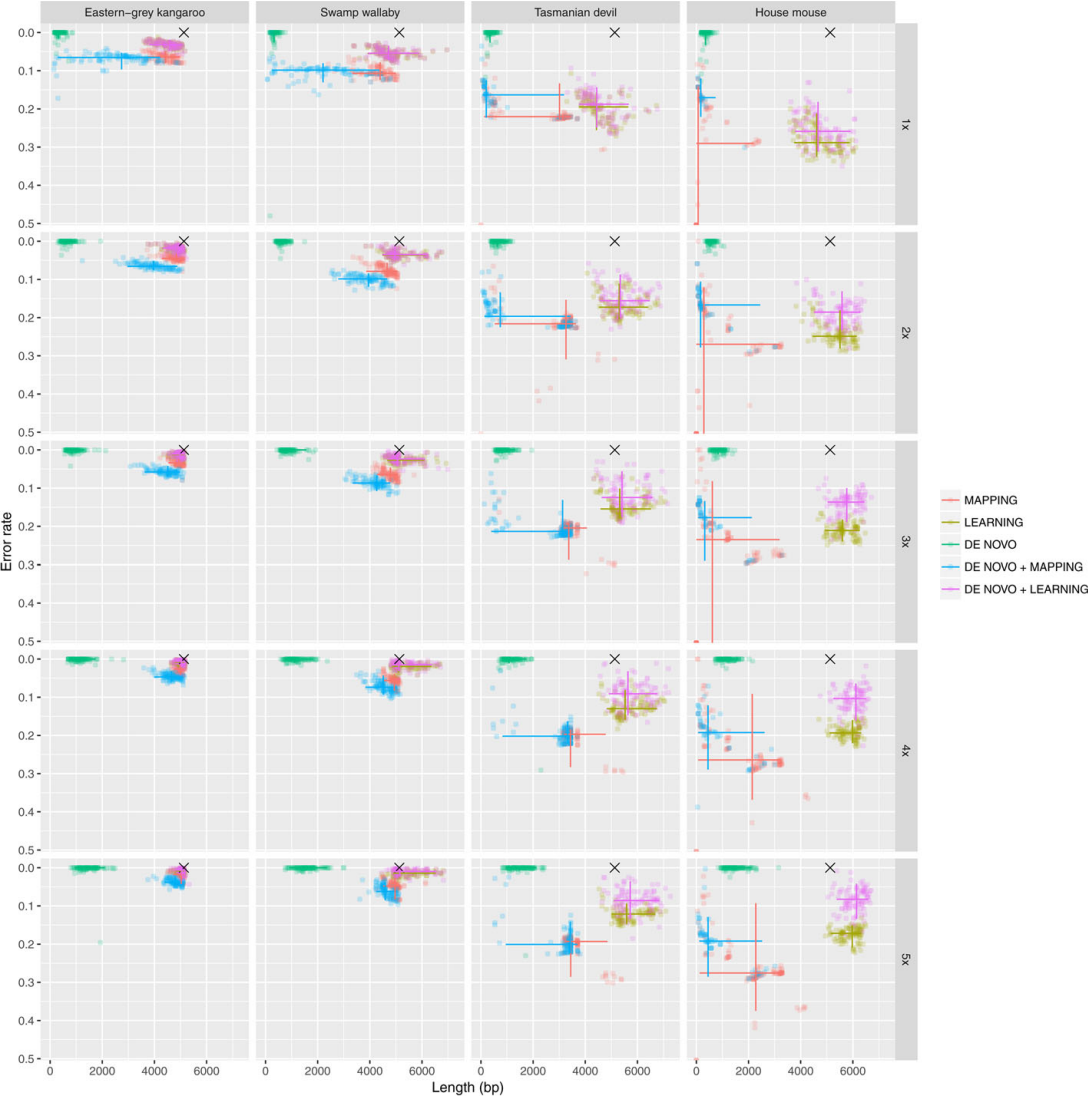
Number of errors and length in nucleotide of the reconstructed amplicon for each bioinformatic pipeline and simulation settings. The 95% intervals are shown as solid lines for each method along both dimensions (reconstructed amplicon length and error rate)

**Fig. 3:**
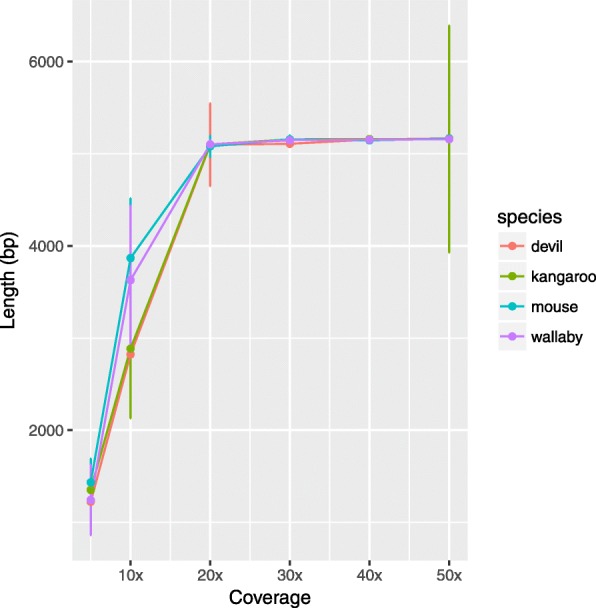
With more than 20× coverage, the de Bruijn graph assembly is able to reconstruct the expected amplicon length (5,130bp)

**Fig. 4:**
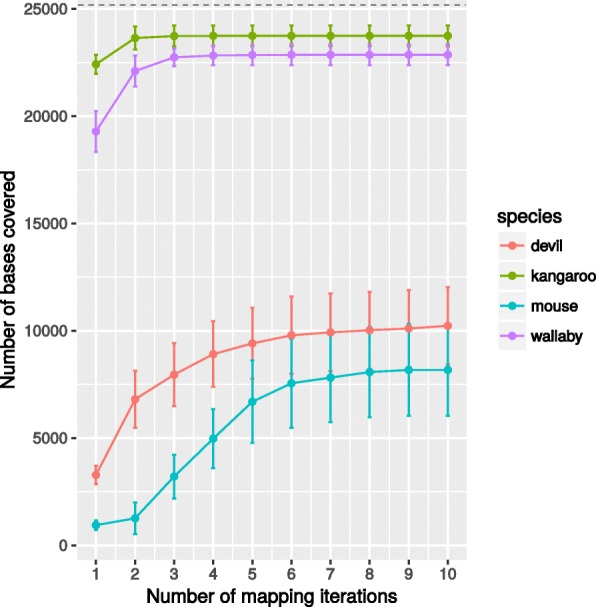
Increasing the number of mapping iteration of the same reads does improve the number of aligned reads, measured as number of bases covered, but only to a limited extend. The short-reads originate from an amplicon of length 5,130bp with 5× coverage, therefore the expected number of bases covered is ∼25, 000 (dashed line)

**Fig. 5:**
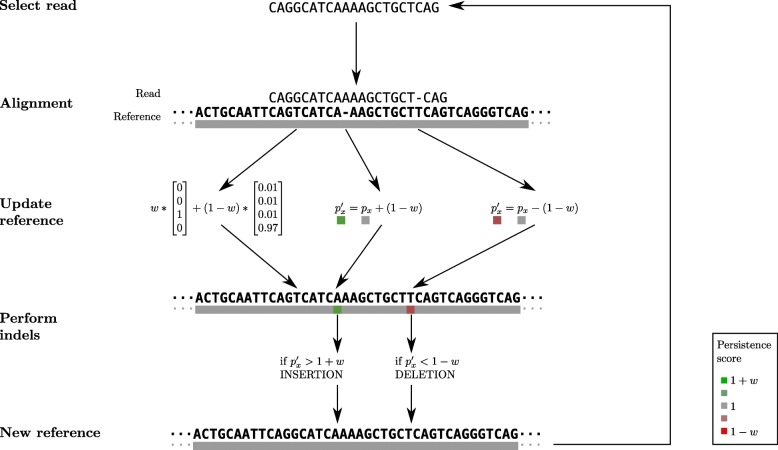
Overview of the algorithm. Reads are taken in random order and iteratively aligned to the reference. After each alignment, the reference sequence is updated according to the learning rate *w*, which is proportional to the normalised edit distance between the read and the reference. In this case, there is one substitution between the reference of the read; the read has a G with Phred quality score of 15 while the reference is *T*. One deletion and one insertion are treated thanks to a persistence vector. The persistence value p• indicates the tendency of a base to be inserted or deleted at each position in the reference. This value can trigger indels update in the reference when it goes beyond a threshold

**Fig. 6:**
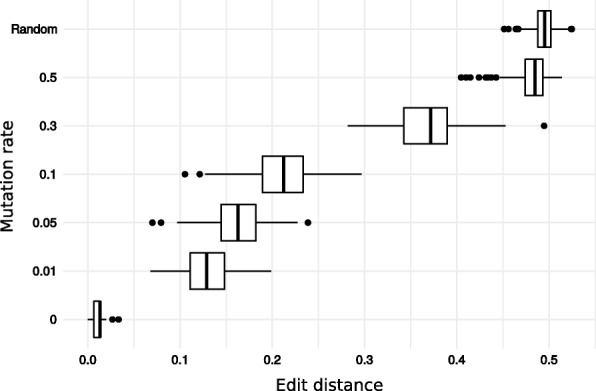
Distribution of the normalised edit distance between reads and increasingly distant reference sequences. The mutation rate of the reference sequence is indicated on the y-axis. The top row (Random) shows the distribution of the edit distance when reads were aligned to randomly generated nucleotide sequences. For the lowest row, the reads were aligned to their original sequence and the departure from 0 of the edit distance only results from the simulated sequencing errors

**Fig. 7:**
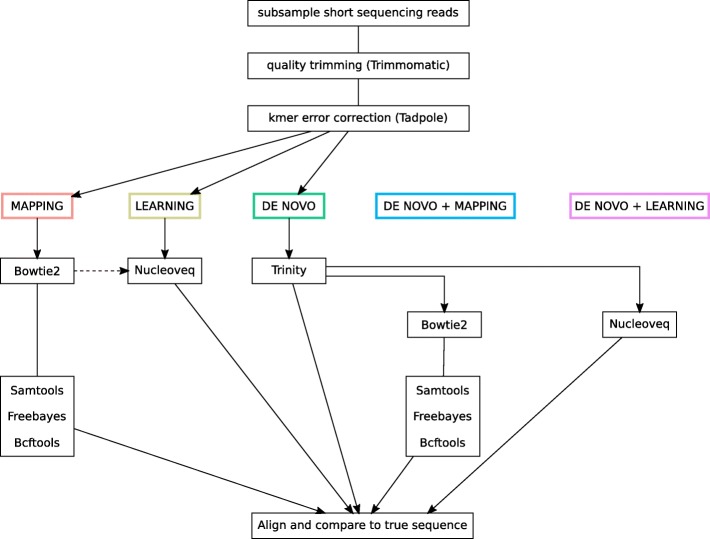
Five bioinformatic pipelines for assembly. Dashed-line: it is possible to pass a priori mapping position of the reads to Nucleoveq to decrease memory requirements and speed up computation (option not used in the reported comparisons)

Incorrect:

Table 1 The four different reference sequences used to guide the reconstruction of the western-grey kangaroo mitochondrial amplicon from short sequencing reads.

Correct:

Table 1 The four different reference sequences used to guide the reconstruction of the western-grey kangaroo mitochondrial amplicon from short sequencing reads. For each circular mitochondrial genome, the genome coordinates of the extracted region are indicated as well as its length. The percentage identity to the western-grey amplicon is calculated on the homologous regions only, i.e. the non-aligned sections at the beginning and the end of the alignment are not taken into account.

In this correction article the figures are shown correct with the correct caption of Table 1.
